# Identification and analysis of mitochondria-related central genes in steroid-induced osteonecrosis of the femoral head, along with drug prediction

**DOI:** 10.3389/fendo.2024.1341366

**Published:** 2024-02-07

**Authors:** Zheru Ma, Jing Sun, Qi Jiang, Yao Zhao, Haozhuo Jiang, Peng Sun, Wei Feng

**Affiliations:** ^1^ Department of Bone and Joint Surgery, Orthopaedic Center, The First Hospital of Jilin University, Chang chun, China; ^2^ Department of Otolaryngology Head and Neck Surgery, The First Hospital of Jilin University, Changchun, Jilin, China; ^3^ Department of Respiratory Medicine, The First Hospital of Jilin University, Changchun, Jilin, China

**Keywords:** steroid-induced femoral head necrosis, weighted gene co-expression network analysis, differentially expressed genes, mitochondrial-related genes, immune cell infiltration, diagnostic biomarkers, bioinformatics analysis, DrugBank

## Abstract

**Purpose:**

Steroid-induced osteonecrosis of the femoral head (SONFH) is a refractory orthopedic hip joint disease that primarily affects middle-aged and young individuals. SONFH may be caused by ischemia and hypoxia of the femoral head, where mitochondria play a crucial role in oxidative reactions. Currently, there is limited literature on whether mitochondria are involved in the progression of SONFH. Here, we aim to identify and validate key potential mitochondrial-related genes in SONFH through bioinformatics analysis. This study aims to provide initial evidence that mitochondria play a role in the progression of SONFH and further elucidate the mechanisms of mitochondria in SONFH.

**Methods:**

The GSE123568 mRNA expression profile dataset includes 10 non-SONFH (non-steroid-induced osteonecrosis of the femoral head) samples and 30 SONFH samples. The GSE74089 mRNA expression profile dataset includes 4 healthy samples and 4 samples with ischemic necrosis of the femoral head. Both datasets were downloaded from the Gene Expression Omnibus (GEO) database. The mitochondrial-related genes are derived from MitoCarta3.0, which includes data for all 1136 human genes with high confidence in mitochondrial localization based on integrated proteomics, computational, and microscopy approaches. By intersecting the GSE123568 and GSE74089 datasets with a set of mitochondrial-related genes, we screened for mitochondrial-related genes involved in SONFH. Subsequently, we used the good Samples Genes method in R language to remove outlier genes and samples in the GSE123568 dataset. We further used WGCNA to construct a scale-free co-expression network and selected the hub gene set with the highest connectivity. We then intersected this gene set with the previously identified mitochondrial-related genes to select the genes with the highest correlation. A total of 7 mitochondrial-related genes were selected. Next, we performed Gene Ontology (GO) and Kyoto Encyclopedia of Genes and Genomes (KEGG) pathway enrichment analysis on the selected mitochondrial-related genes using R software. Furthermore, we performed protein network analysis on the differentially expressed proteins encoded by the mitochondrial genes using STRING. We used the GSEA software to group the genes within the gene set in the GSE123568 dataset based on their coordinated changes and evaluate their impact on phenotype changes. Subsequently, we grouped the samples based on the 7 selected mitochondrial-related genes using R software and observed the differences in immune cell infiltration between the groups. Finally, we evaluated the prognostic significance of these features in the two datasets, consisting of a total of 48 samples, by integrating disease status and the 7 gene features using the cox method in the survival R package. We performed ROC analysis using the roc function in the pROC package and evaluated the AUC and confidence intervals using the ci function to obtain the final AUC results.

**Results:**

Identification and analysis of 7 intersecting DEGs (differentially expressed genes) were obtained among peripheral blood, cartilage samples, hub genes, and mitochondrial-related genes. These 7 DEGs include FTH1, LACTB, PDK3, RAB5IF, SOD2, and SQOR, all of which are upregulated genes with no intersection in the downregulated gene set. Subsequently, GO and KEGG pathway enrichment analysis revealed that the upregulated DEGs are primarily involved in processes such as oxidative stress, release of cytochrome C from mitochondria, negative regulation of intrinsic apoptotic signaling pathway, cell apoptosis, mitochondrial metabolism, p53 signaling pathway, and NK cell-mediated cytotoxicity. GSEA also revealed enriched pathways associated with hub genes. Finally, the diagnostic value of these key genes for hormone-related ischemic necrosis of the femoral head (SONFH) was confirmed using ROC curves.

**Conclusion:**

BID, FTH1, LACTB, PDK3, RAB5IF, SOD2, and SQOR may serve as potential diagnostic mitochondrial-related biomarkers for SONFH. Additionally, they hold research value in investigating the involvement of mitochondria in the pathogenesis of ischemic necrosis of the femoral head.

## Introduction

Hormone-related osteonecrosis of the femoral head (SONFH) is a chronic and refractory orthopedic disease, which primarily affects the blood supply to the femoral head, leading to ischemia, necrosis, and eventual collapse of the bone tissue (1, 2). SONFH is more common in middle-aged adults, and without effective systemic treatment, approximately 80% of SONFH patients experience femoral head collapse within 1 to 4 years, requiring total hip replacement surgery (3). High-dose hormone therapy, particularly long-term use of corticosteroids such as prednisone, is one of the major risk factors for inducing SONFH. Mitochondria, as the energy factories of cells, play a crucial role in maintaining cellular function and survival (4). Currently, the relationship between mitochondria and hormone-related ischemic necrosis of the femoral head mainly manifests in the following aspects: 1. Mitochondrial dysfunction: Hormone therapy may lead to impaired mitochondrial function, affecting mitochondrial oxidative phosphorylation and energy production. This may result in cellular energy deficiency, ultimately leading to bone cell death and ischemic necrosis of the femoral head. 2.Oxidative stress: Hormone therapy may cause mitochondria to produce excessive reactive oxygen species (ROS), triggering oxidative stress (5). Oxidative stress can damage cell membranes, proteins, and nucleic acids, affecting normal cell function. In bone cells, oxidative stress may induce cell apoptosis, further exacerbating the development of ischemic necrosis of the femoral head (6). 3.Calcium ion homeostasis imbalance: Hormone therapy may affect the calcium ion homeostasis of mitochondria, leading to disruption of calcium ion balance within cells. Calcium ions play a crucial role in cell signaling and bone metabolism, and an imbalance in calcium ion homeostasis may impair bone cell function and contribute to ischemic necrosis of the femoral head (7). 4.Mitochondria-regulated cell apoptosis: Mitochondria play a key role in regulating cell apoptosis, and hormone therapy may activate mitochondrial pathways of cell apoptosis. Excessive cell apoptosis may result in bone tissue loss and ischemic necrosis of the femoral head (6, 8–10). 5.Autophagy: Mitochondria maintain their own quality and function through the process of autophagy. Hormone therapy may affect mitochondrial autophagy, further exacerbating mitochondrial dysfunction and cellular damage (11, 12). In summary, mitochondria play a crucial role in hormone-related ischemic necrosis of the femoral head (13). Hormone therapy may lead to mitochondrial dysfunction, oxidative stress, calcium ion homeostasis imbalance, cell apoptosis, and autophagy disruption, ultimately contributing to the development of ischemic necrosis of the femoral head. Recent studies on mitochondria-related ferroptosis have introduced new directions in the research of mitochondria and ischemic necrosis of the femoral head (14). Therefore, further in-depth research is needed to elucidate the mechanisms of action of mitochondria in hormone-related ischemic necrosis of the femoral head, providing new targets for the prevention and treatment of such diseases. Here, we utilize bioinformatics methods to explore the gene-level connection between hormone-related ischemic necrosis of the femoral head and mitochondria, aiming to validate existing relevant conclusions and investigate the underlying mechanisms associated with ischemic necrosis of the femoral head.

## Materials and methods

Microarray data source: The gene expression data utilized in this study were obtained from the NCBI Gene Expression Omnibus (GEO), specifically the GSE123568 and GSE74089 datasets, which are associated with hormone-related ischemic necrosis of the femoral head (SONFH). The GSE123568 dataset comprises mRNA expression profiles from 10 non-SONFH samples (after corticosteroid administration) and 30 SONFH samples. On the other hand, the GSE74089 dataset includes mRNA expression profiles from 4 healthy samples and 4 samples of hip cartilage affected by femoral head ischemic necrosis. These datasets were downloaded from the GEO database using the GPL15207 and GPL13497 platforms. For further details, please refer to **Table 1**. To investigate mitochondrial involvement, the MitoCarta 3.0 database was utilized, which encompasses data on 1136 human genes with high confidence in mitochondrial localization(**Supplementary Table 1**). These gene annotations were determined based on a combination of proteomics, computational analysis, and microscopy techniques.

**Table 1:** Two datasets used for gene expression profiles analysis in SONFH.

GEO ID	Author	Platform	SONFH group	Control group	Species	Type	Year	Omics
**GSE123568**	Zhang Y	GPL15207	30	10	Homo sapien	Peripheral serum	2019	mRNA
**GSE74089**	Ruiyu L	GPL13497	4	4	Homo sapien	Hip articular cartilage	2016	mRNA

The gene datasets were subjected to batch processing: For the merging of the GSE123568 and GSE74089 datasets, we employed the inSilicoMerging R software package, which facilitated the integration of the datasets. To further enhance the quality of the data, we applied the method proposed by Johnson et al. (Empirical Bayes: Adjusting batch effects in microarray expression data using empirical Bayes methods, DOI: 10.1093/biostatistics/kxj037) to effectively eliminate any batch effects. This step ensured that the subsequent analysis was performed on the processed samples and genes, guaranteeing reliable results.

Differential gene expression analysis was conducted using the “limma” software package in R: Genes meeting the criteria of p_adj <0.01 and abs |log2 fold change (FC)|> log_2_1.5 were considered as differentially expressed genes (DEGs), indicating their potential significance in the study. To visually represent the results, heatmaps and volcano plots were generated using the “pheatmap” and “ggplot2” packages, respectively. These graphical representations provide a comprehensive overview of the DEGs and their expression patterns.

Preliminary screening of differentially expressed genes (DEGs) related to mitochondria: We performed an intersection analysis between the DEGs identified in the limma differential analysis of GES123568 and GSE74089 datasets, specifically in the upregulated and downregulated gene sets, with the MitoCarta 3.0 mitochondrial dataset. This analysis aimed to identify DEGs that are specifically associated with mitochondria. By intersecting the DEGs with the MitoCarta 3.0 dataset, we obtained a preliminary list of differentially expressed genes that are potentially involved in mitochondrial functions and processes.

Weighted Gene Co-expression Network Analysis (WGCNA): In order to explore the relationships between gene co-expression and phenotypic traits, we utilized the “WGCNA” package in R software to construct a comprehensive gene co-expression network. To ensure the reliability of the network, we initially constructed a clustering tree and removed any outliers based on the results. Subsequently, we selected the top 5000 genes with a median absolute deviation (MAD) greater than 5. The correlation coefficients between gene pairs were computed to construct a similarity matrix. To establish a scale-free network, we carefully determined an appropriate soft threshold to transform the similarity matrix into an adjacency matrix. Following this, we generated a topological overlap matrix (TOM) to evaluate the average network connectivity of each gene. By employing the blockwiseModules function with specific parameters such as minModuleSize and mergeCutHeight, we employed a dynamic tree-cutting method to assign genes with similar expression patterns into distinct modules. Each module was visually represented by a unique color, with genes in the gray module indicating those that could not be assigned to any specific module. To summarize the expression profiles within each module, we calculated the first principal component known as the module eigengene (ME). The ME was utilized to assess the associations between modules and phenotypic traits. The module with the highest absolute correlation coefficient was identified as the key module for further analysis. Additionally, we measured the module membership (MM), which represents the correlation between a gene’s expression and the ME of a module, indicating the gene’s relevance to that particular module. Moreover, the gene significance (GS) was determined by assessing the correlation between a gene’s expression and the phenotype, providing insights into the gene’s association with the phenotype.

Identification of Hub Genes: In order to identify key genes associated with mitochondrial and hormonal factors in hormone-related femoral head ischemic necrosis (SONFH), we employed the “VennDiagram” package in R software to determine the intersection of hub genes obtained from the Weighted Gene Co-expression Network Analysis (WGCNA) and the initially screened set of genes related to mitochondria. To visualize the differential expression of hub genes between SONFH and non-SONFH samples, we utilized violin plots. Statistical hypothesis tests, including t-tests and Mann-Whitney U tests, were conducted to evaluate the significance of the observed differences. The choice of test depended on the distribution of the data, with the t-test used for normally distributed data and the Mann-Whitney U test employed for non-normally distributed data. Significance was defined as p < 0.05.

Enrichment Analysis: To investigate the biological mechanisms underlying mitochondrial-related hub genes associated with SONFH, functional enrichment analysis was conducted. Initially, we analyzed the Gene Ontology (GO) biological processes (BP) associated with these genes, and the final results were visualized using chord diagrams with the “GOplot” package in R software. Subsequently, gene set enrichment analysis (GSEA) was performed to reveal the specific functions of each gene. All of these analyses were conducted using the “clusterProfiler” package in R software, with a significance threshold set at p_adj < 0.05.

Protein-Protein Interaction (PPI) Network Analysis: To gain a deeper understanding of the intricate relationships among differentially expressed autophagy-related genes in SONFH, we conducted a Protein-Protein Interaction (PPI) network analysis. We utilized the Search Tool for the Retrieval of Interacting Genes (STRING, version: 11.0, https://stringdb.org/) and Cytoscape software (version: 3.7.2, http://Cytoscape.org/) to analyze and construct the PPI network. A combination score threshold of medium confidence (>0.4) was set for the interactions, and isolated nodes were discarded.

Clustering based on Key Genes and Immune Infiltration Analysis: Considering the potential presence of immune cell infiltration in SONFH, we aimed to validate the relationship between peripheral immune cells and mitochondria. To achieve this, we conducted clustering analysis using ConsensusClusterPlus (Wilkerson and Hayes, 2010, ConsensusClusterPlus: A class discovery tool with confidence assessments and item tracking, DOI:10.1093/bioinformatics/btq170). The pam clustering method with a 1-Pearson correlation distance was employed, and 80% of the samples were resampled 10 times to determine the optimal number of clusters based on empirical cumulative distribution function plots. The clustering results based on key genes were used to group SONFH samples in GSE123568. To assess immune cell infiltration in the microenvironment, we utilized CIBERSORT, which incorporates 547 biomarkers and 22 human immune cell subtypes, including plasma cells, B cells, T cells, and myeloid cells. CIBERSORT employs linear support vector regression principles for deconvolution analysis of immune cell expression profiles. In this study, expression data from GSE132903 were utilized, and the relative proportions of the 22 immune cell types were quantified in each sample. Furthermore, Spearman correlation analysis was performed to examine the correlation between hub genes and immune infiltration, as well as immune factors. This analysis was conducted using the “psych” package in R software, and the results were visualized as a heatmap.

Receiver Operating Characteristic (ROC) Curve Analysis: Logistic regression is a generalized linear regression model commonly used for automated disease diagnosis. In this study, logistic regression with two response variables was employed. A response variable of 1 indicated SONFH samples, while a response variable of 0 indicated noSONFH samples. Stepwise regression analysis was utilized to eliminate non-significant factors associated with the response variable, resulting in a simplified model with only statistically significant factors. The stepwise regression process iteratively added or removed variables in order to minimize the statistical value of the Akaike information criterion (AIC). Subsequently, logistic regression was applied to establish the relationship between these significant factors and the response variable. The diagnostic performance of the model was evaluated using receiver operating characteristic (ROC) curves and the corresponding area under the curve (AUC). The AUC provides a measure of the model’s diagnostic accuracy.

We employed the aforementioned approach to assess the prognostic significance of these features in 40 samples from GSE123568 and GSE74089. ROC analysis was conducted using the pROC package (version 1.17.0.1) in the R software to obtain the AUC.

Drug Targeting Hub Genes from DrugBank: The drugs targeting hub genes were retrieved from the DrugBank database. DrugBank is a comprehensive chemical and bioinformatics repository that houses detailed information about various drugs and their corresponding targets. With a collection of over 7,800 drugs, it encompasses a wide range of substances, including nutritional supplements, investigational drugs, FDA-approved small molecule drugs, and FDA-approved biotech drugs (Wishart et al., 2018). Additionally, DrugBank offers a vast array of SNP drugs that can be utilized for pharmacogenomic research.

Statistical Analysis: All analyses were performed using R software. The choice between t-test and Mann-Whitney U test was determined based on the normality distribution of the data. Significance was typically defined as p < 0.05.

## Results

### Identification of hub genes

**Figure 1:**
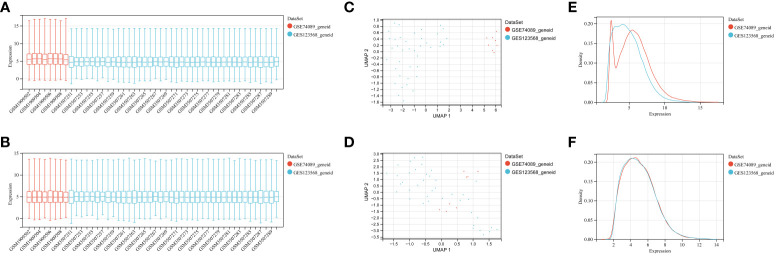
GEO data batch correction. **(A)** Statistical analysis of gene expression levels in the pre-batch processed dataset. **(B)** Statistical analysis of gene expression levels in the integrated dataset after batch correction. **(C)** Umap distribution between datasets before batch correction. **(D)** Umap distribution between integrated datasets after batch correction. **(E)** Comparison of density distributions in the pre-batch processed dataset. **(F)** Comparison of density distributions in the dataset after batch correction.

**Figure 2:**
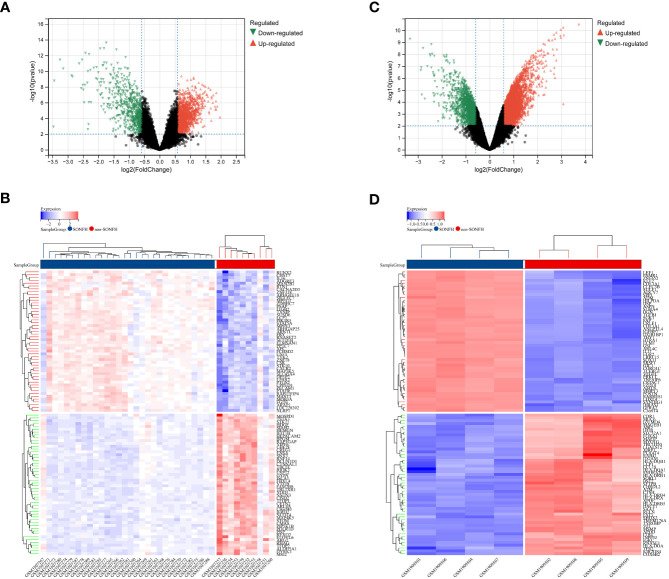
Differentially expressed genes (DEGs) related to GC-related femoral head avascular necrosis. **(A)** Volcano plot of DEGs in GSE123568 associated with femoral head necrosis. The x-axis represents log2FoldChange, and the y-axis represents -log10(adjusted P-value). Red nodes indicate upregulated DEGs, green nodes indicate downregulated DEGs, and black nodes represent genes with no significant differential expression. **(B)** Heatmap of expression levels of DEGs in GSE123568 associated with femoral head necrosis: blue represents disease samples, red represents normal control samples, red represents genes with high expression, and blue represents genes with low expression. **(C)** Volcano plot of DEGs in GSE74089 associated with femoral head necrosis. The x-axis represents log2FoldChange, and the y-axis represents -log10(adjusted P-value). Red nodes indicate upregulated DEGs, green nodes indicate downregulated DEGs, and black nodes represent genes with no significant differential expression. **(D)** Heatmap of expression levels of DEGs in GSE74089 associated with femoral head necrosis: blue represents disease samples, red represents normal control samples, red represents genes with high expression, and blue represents genes with low expression.

**Figure 3:**
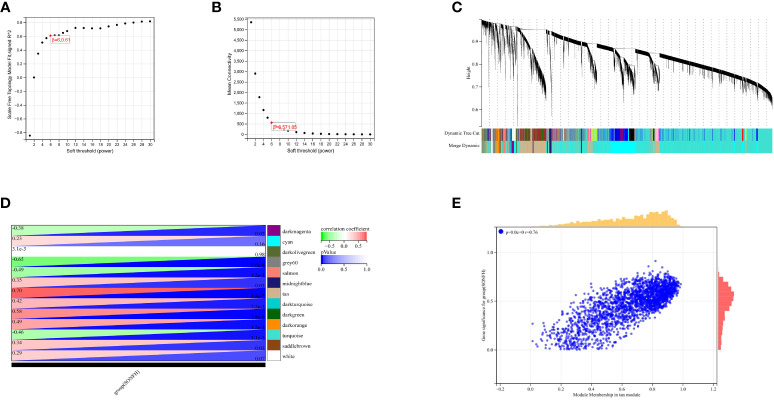
Results of WGCNA. **(A)** Fit indices of scale-free topology model under different soft thresholding powers. **(B)** Average connectivity values under different soft thresholding powers. **(C)** Gene dendrogram. **(D)** Correlation between different modules and clinical features. Red indicates positive correlation, and blue indicates negative correlation. **(E)** Correlation between module membership and gene importance in the tan module.

**Figure 4:**
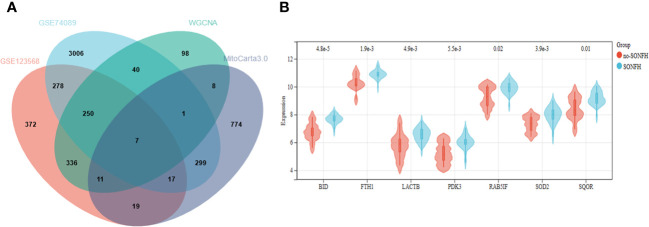
**(A)** Seven hub genes were obtained by taking the intersections of the DEGs. **(B)** Expression of hub genes in the SONFH and Non-SONFH groups of blood samples GSE123568.

To identify genes associated with SONFH, we performed batch correction on two datasets, GSE123568 and GSE74089 (**Figure 1**), excluded outlier genes, and then used the “limma” package in R software to obtain 1,924 and 5,438 differentially expressed genes (DEGs) from GSE123568 and GSE74089, respectively. The selection criteria were set as “p_adj < 0.01 and |logFC| > 0.585” (**Supplementary Table 2**; **Table 2**). These DEGs were visualized in a volcano plot (**Figure 2**). The heatmap of the top 50 DEGs was also generated. After removing outlier samples and filtering genes, a total of 18,654 genes and 40 samples were used to construct a weighted gene co-expression network from GSE123568. When the soft threshold was set to 6, the scale independence reached 0.61, with an average connectivity value of 571.05 (**Figure 3**). By setting a cutting height of 0.25 and a minimum module size of 30, we obtained 13 distinct co-expression modules through dynamic tree cutting (**Figure 3B**). Subsequently, we performed correlation analysis between each module and clinical traits. The tan module showed the highest positive correlation with SONFH (r=0.7, p=4.8e-7), while the turquoise module showed the highest negative correlation with SONFH (r=-0.46, p=3.1e-3; **Figure 3C**). Here, we selected the tan module with the largest absolute correlation coefficient, which included 751 genes(**Supplementary Table 4**), for further analysis. Additionally, the correlation analysis between MM and GS revealed a strong association between these genes and modules as well as phenotypes (cor=0.76, p=0.0e+0; **Figure 3D**). By intersecting the 1,924 DEGs from GSE123568, 5,438 DEGs from GSE74089, 751 genes from the tan module, and 1,136 mitochondria-related genes, we identified 7 mitochondria-related hub genes associated with SONFH (**Figure 4**), including BID, FTH1, LACTB, PDK3, RAB5IF, SOD2, and SQOR. Violin plots showed that these 7 genes were highly expressed in the SONFH group. For a more detailed understanding of the significance and characteristics of each differentially expressed gene, please refer to the comprehensive information provided in **Table 3**.

**Table 2:** Information on drugs targeting these hub genes.

DrugBank ID	Name	Indication/Associated Conditions
**DB03297**	Benzylsulfonic acid	Not Available
**DB04436**	3-Fluoro-L-tyrosine	Not Available
**DB06796**	Mangafodipir	Hepatic Lesions
**DB09221**	Polaprezinc	Peptic ulcer disease dyspepsia
**DB11590**	Thimerosal	Skin disinfection
**DB12756**	TAK-901	Not Available
**DB03758**	Radicicol	Not Available
**DB03760**	Dihydrolipoic Acid	Not Available
**DB09499**	Thiosulfuric acid	prevention of ototoxicity treatment of acute cyanide poisoning Ototoxicity From Cisplatin Chemotherapy
**DB04137**	Guanosine-5’-Triphosphate	Not Available
**DB02467**	L-methionine (S)-S-oxide	Not Available
**DB04315**	Guanosine-5’-Diphosphate	Not Available
**DB00893**	Iron Dextran	Iron Deficiency (ID)
**DB01592**	Iron	Anemia Iron Deficiency (ID) Iron Deficiency Anemia (IDA) Restless Legs Syndrome (RLS) Concomitant myelosuppressive chemotherapy
**DB13995**	Ferric pyrophosphate citrate	Iron Deficiency (ID)
**DB14488**	Ferrous gluconate	Folate deficiency Iron Deficiency (ID) Iron Deficiency Anemia (IDA) Zinc Deficiency
**DB14489**	Ferrous succinate	Iron Deficiency (ID)
**DB14490**	Ferrous ascorbate	Used in preventing and treating iron-deficiency anemia.
**DB14491**	Ferrous fumarate	Folic acid antagonist overdose Iron Deficiency (ID) Iron Deficiency Anemia (IDA)
**DB14501**	Ferrous glycine sulfate	Iron Deficiency (ID)
**DB06784**	Gallium citrate Ga-67	Bronchogenic Carcinoma Hodgkins Disease (HD) Lymphomas NEC Acute inflammatory lesions

**Table 3:** The significance and characteristics of each differentially expressed gene (Gene details from NCBI).

Official Symbol	Official Full Name	Primary source	Gene type	Expression
**BID**	BH3 interacting domain death agonist	HGNC : HGNC:1050	protein coding	Ubiquitous expression in bone marrow (RPKM 13.8), brain (RPKM 9.4) and 24 other tissues
**FTH1**	ferritin heavy chain 1	HGNC : HGNC:3976	protein coding	Ubiquitous expression in colon (RPKM 1259.0), appendix (RPKM 1256.9) and 25 other tissues
**LACTB**	lactamase beta	HGNC : HGNC:16468	protein coding	Ubiquitous expression in bone marrow (RPKM 9.0), adrenal (RPKM 7.3) and 25 other tissues
**PDK3**	pyruvate dehydrogenase kinase 3	HGNC : HGNC:8811	protein coding	Ubiquitous expression in brain (RPKM 5.9), testis (RPKM 4.2) and 25 other tissues
**RAB5IF**	RAB5 interacting factor	HGNC : HGNC:15870	protein coding	Ubiquitous expression in bone marrow (RPKM 56.7), esophagus (RPKM 45.4) and 25 other tissues
**SOD2**	superoxide dismutase 2	HGNC : HGNC:11180	protein coding	Ubiquitous expression in appendix (RPKM 186.4), bone marrow (RPKM 85.8) and 23 other tissues
**SQOR**	sulfide quinone oxidoreductase	HGNC : HGNC:20390	protein coding	Broad expression in colon (RPKM 63.6), esophagus (RPKM 32.6) and 21 other tissues

### Enrichment of hub genes in biological processes and pathways

**Figure 5:**
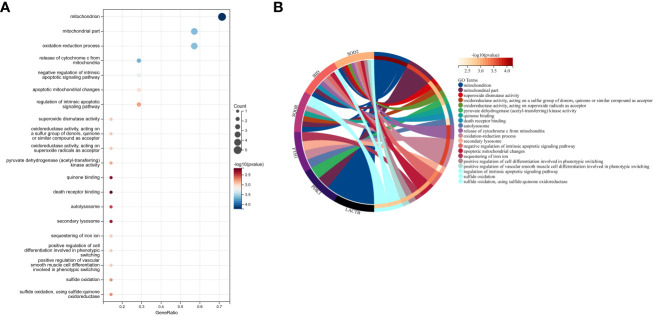
Performing GO functional enrichment analysis for 7 key mitochondrial-related genes in SONFH. **(A)** Bubble plot showing the top 15 enriched functions for the 7 target genes. **(B)** Chord plot representing the relationship between the top 15 enriched functions and the targets, with node colors arranged in descending order of logFC values from red to blue. Genes are sorted based on logFC values.

**Figure 6:**
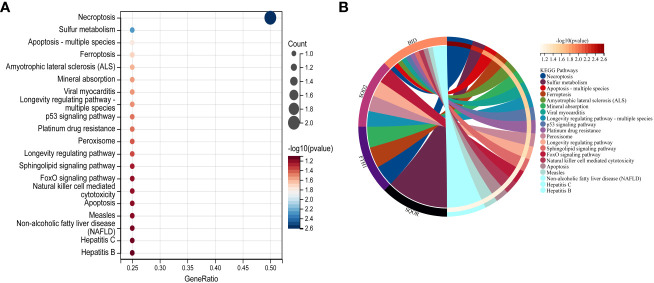
Performing KEGG pathway analysis for 7 key mitochondrial-related genes in SONFH. **(A)** Bubble plot showing the top 15 pathways enriched for the 7 target genes. **(B)** Chord plot representing the relationship between the top 15 pathways and the targets. Node colors are arranged in descending order of logFC values from red to blue. Genes are sorted based on logFC values.

**Figure 7:**
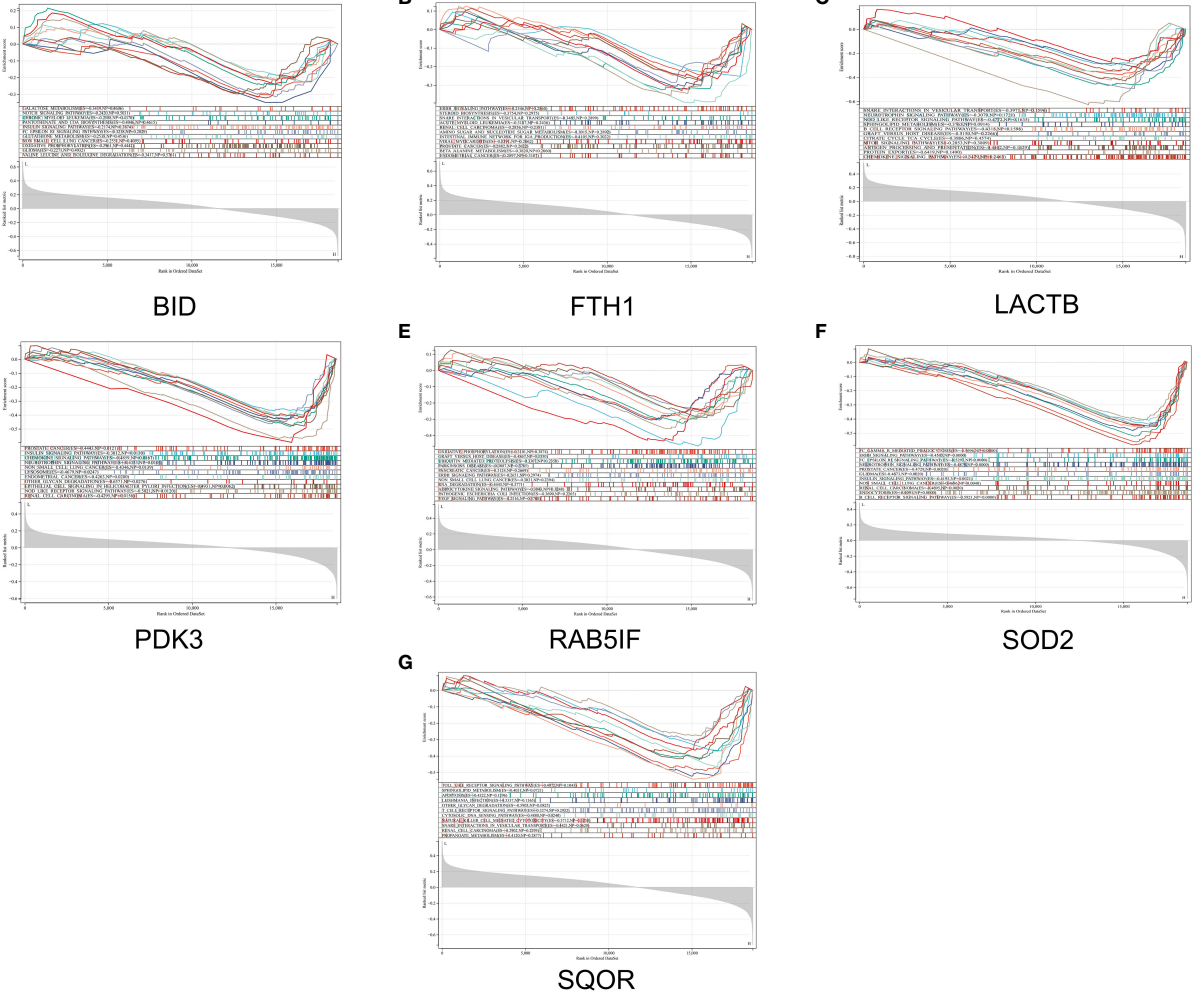
GSEA reveals enriched pathways of hub genes. **(A)** BID, **(B)** FTH1, **(C)** LACTB, **(D)** PDK3, **(E)** RAB5IF, **(F)** SOD2, **(G)** SQOR.

In this study, we utilized the DAVID database for GO and KEGG pathway enrichment analysis of the upregulated genes. The GO analysis of the upregulated DEGs (**Figure 5**) revealed that the biological processes (BP) were mainly enriched in processes such as oxidation-reduction, release of cytochrome C from mitochondria, negative regulation of intrinsic apoptotic signaling pathway, and superoxide dismutase activity. The cellular components (CC) were primarily associated with mitochondria and autophagosomes. The molecular functions (MF) showed regulation of acetyl-CoA acetyltransferase activity. The KEGG pathway enrichment analysis of the upregulated genes (**Figure 6**) indicated their involvement in pathways related to necroptosis, sulfur metabolism, apoptosis, rust disease, p53 signaling pathway, and NK cell-mediated cytotoxicity. GSEA revealed enriched pathways for the hub genes (**Figure 7**). The GSEA analysis demonstrated their association with various signaling pathways, including TGF-β signaling pathway, insulin signaling pathway, vascular endothelial growth factor signaling pathway, ErbB signaling pathway, B cell receptor signaling pathway, T cell receptor signaling pathway, oxidative phosphorylation, RNA degradation, apoptosis, NK cell-mediated cytotoxicity, prostate cancer, glioma, non-small cell lung cancer, and protein export-related pathways.

### Protein-protein interaction network analysis

**Figure 8:**
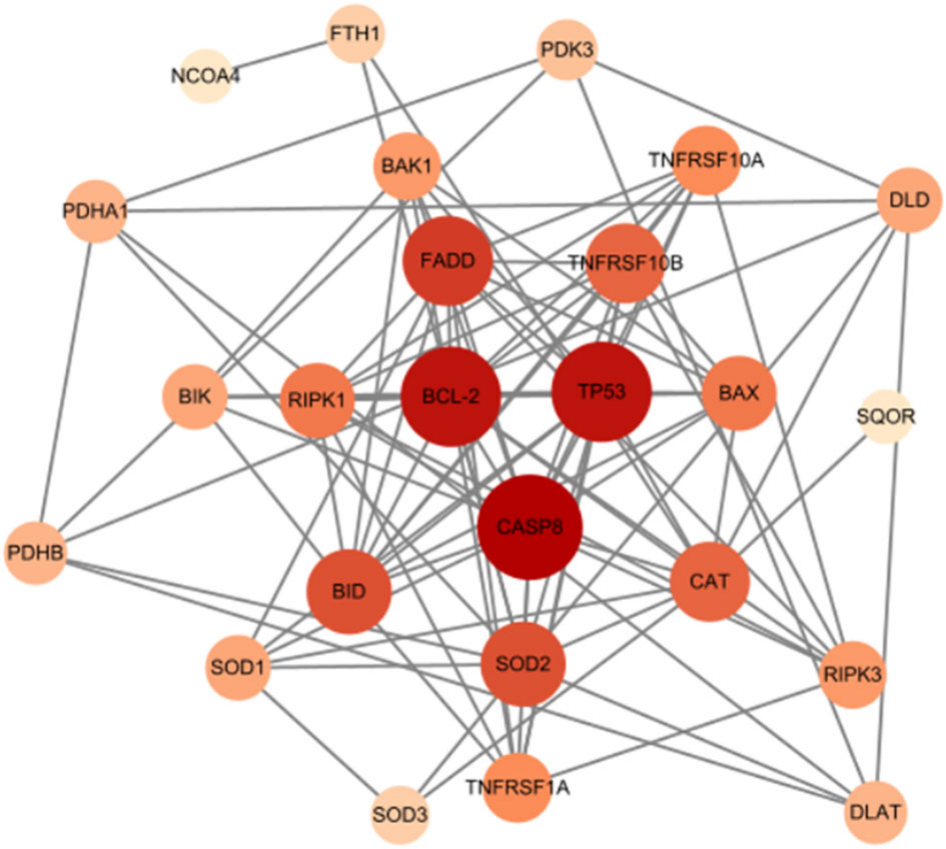
Mitochondrial-related gene protein-protein interaction (PPI) network.

To explore the intricate interactions among the 7 differentially expressed mitochondria-related genes, a PPI analysis was conducted using the STRING database. This analysis aimed to investigate the interactions between these mitochondria-related genes, and the number of interactions for each gene was visualized using Cytoscape, based on their relevance. As shown in **Figure 8**, LACTB and RAB5IF were excluded as independent nodes. The results revealed that the associated genes were involved in the BCL-2/BAX apoptotic pathway and the ROS/JNK/c-Jun signaling pathway, further highlighting the significant role of cell apoptosis in glucocorticoid-induced osteonecrosis of the femoral head.

### Clustering and immune infiltration analysis based on key genes

**Figure 9:**
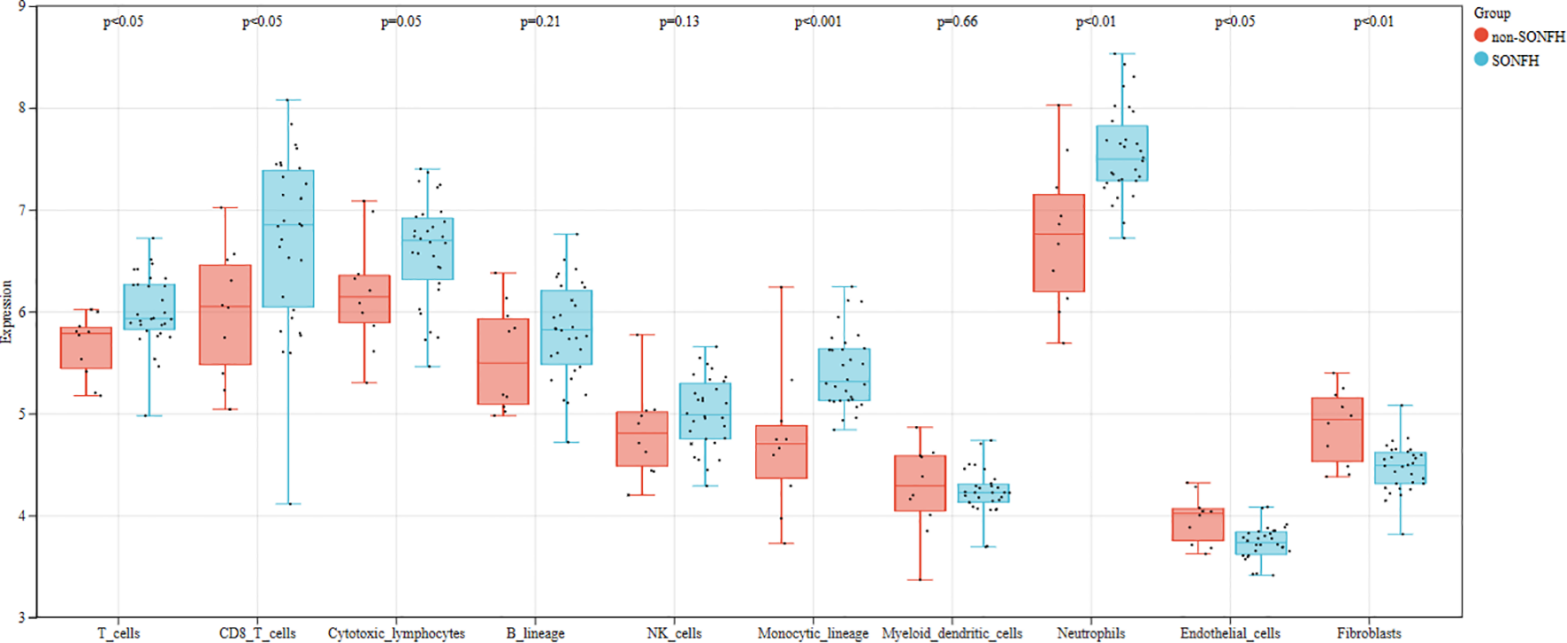
Differences in immune cell infiltration between the SONFH group and the non-SONFH group.

**Figure 10:**
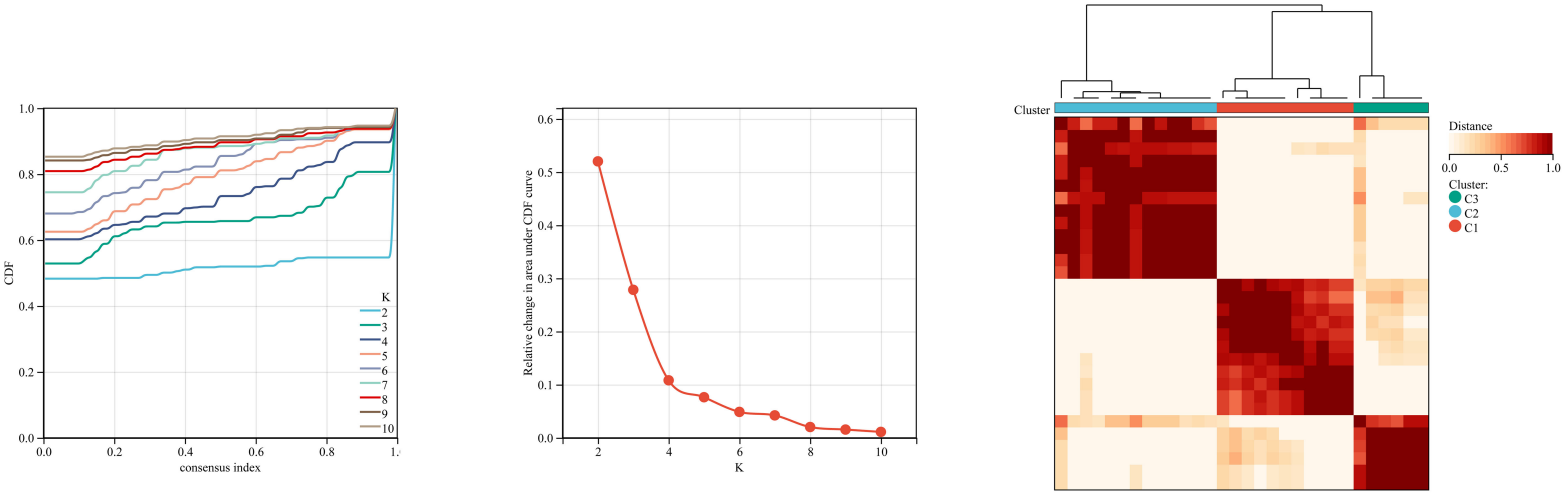
Using ConsensusClusterPlus for clustering analysis, the SONFH group was divided into three clusters based on key mitochondrial-related genes.

**Figure 11:**
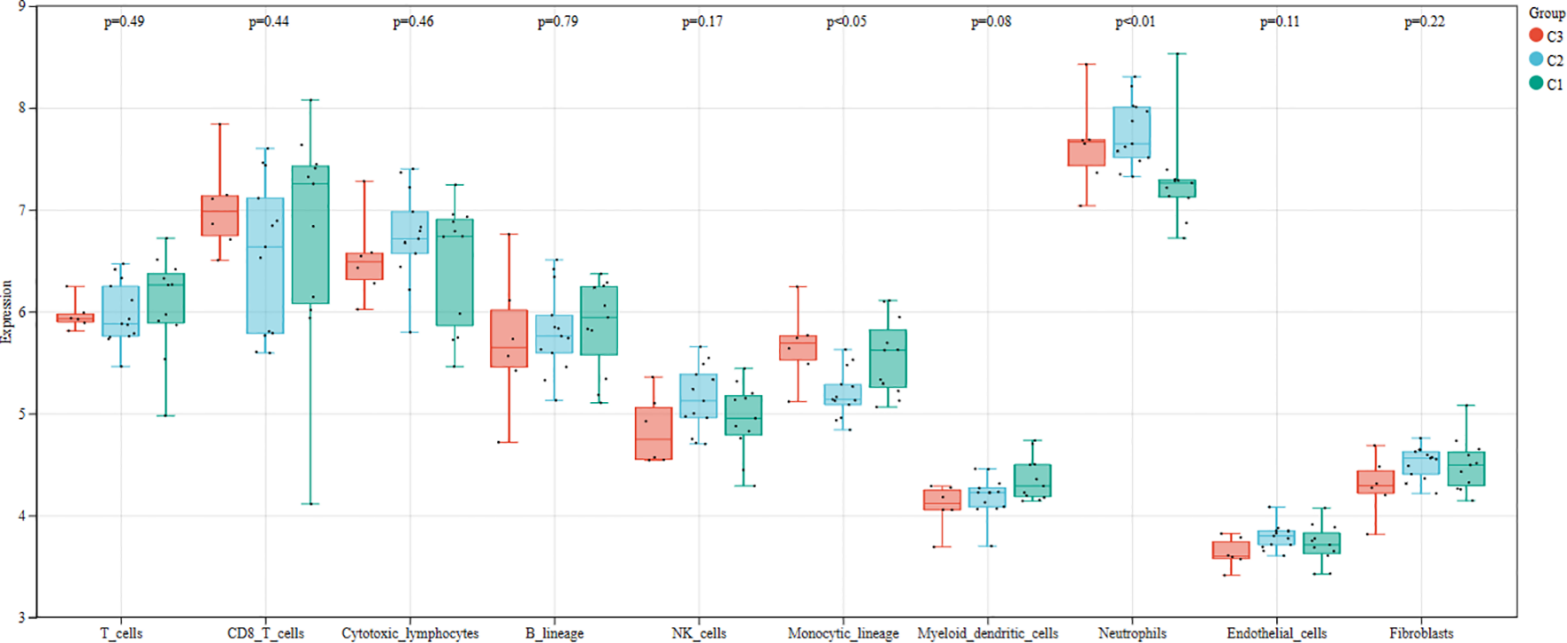
Differences in immune cell infiltration among different clusters within the SONFH group.

We first used the CIBERSORT method to compare the immune cell infiltration between the SONFH and non-SONFH groups. According to the results, we found that the infiltration of T cells, CD8 T cells, cytotoxic lymphocytes, monocytes, neutrophils, endothelial cells, and fibroblasts was significantly increased in the SONFH group (**Figure 9**). Next, based on the expression levels of DEGs, the clustering stability was optimal when k = 3, with k ranging from 2 to 10 (**Figure 10**). Thirty SONFH patients were divided into three subtypes: Cluster1 (n = 8), Cluster2 (n = 10), and Cluster3 (n = 12). To investigate the relationship between subtypes and immune cell infiltration in the peripheral blood of SONFH patients, we evaluated the relative levels of immune cells in the three subtypes using the CIBERSORT method (**Figure 11**). Cluster2 patients showed a significant increase in T cell and CD8 T cell infiltration, while neutrophil infiltration was lower. Cluster3 patients exhibited a significant increase in monocytes and a marked decrease in CD8 T cells. These results suggest that hub genes may play an important role in the immune microenvironment.

### Receiver operating characteristic curve analysis

**Figure 12:**
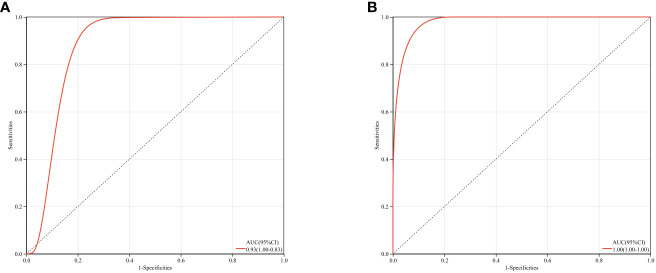
ROC curves and corresponding AUC values for two expression datasets. **(A)** Blood samples from GSE123568. **(B)** Cartilage samples from GSE74089.

Based on GSE123568 and GSE74089, a multi-gene prediction model was established using logistic regression algorithm. Stepwise regression analysis was conducted to select all seven previously obtained genes for the optimal model. The results demonstrated that the prediction model constructed from these seven genes exhibited good diagnostic performance, with an area under the curve (AUC) of 0.93 and 1 (**Figure 12**). These findings suggest that these seven genes may have good diagnostic value.

### Drugs from the DrugBank

**Figure 13:**
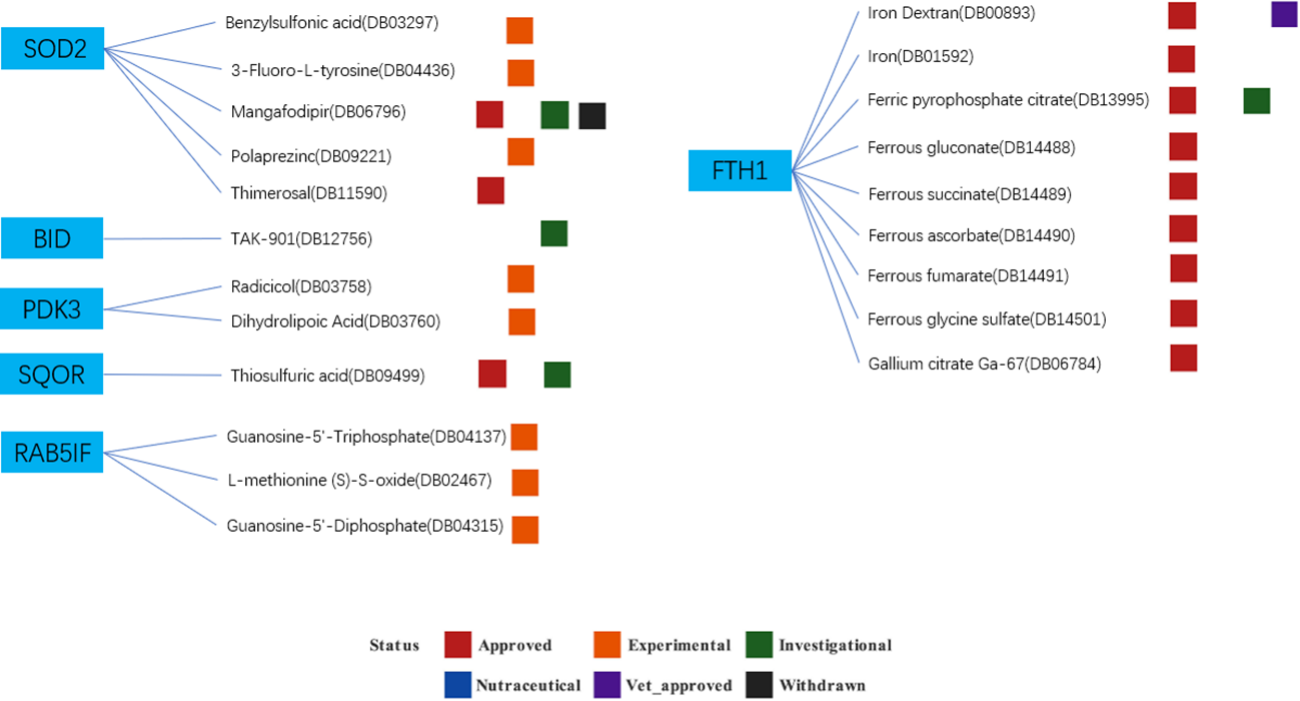
Drugs targeting these seven hub genes obtained from the DrugBank database. Drug statuses, including approved, experimental, investigational,nutraceutical, vet_approved and withdrawn, are indicated by colored squares. Drug types, including agonist, binder, cofactor, and inhibitor, are indicated by colored circles.

Based on the drug and target information from the DrugBank database, twenty-two drugs targeting these seven hub genes were identified (**Figure 13**). Among these drugs, eleven are approved, eight are experimental, two are investigational, and one has been withdrawn. For specific drug actions, please refer to **Table 2**.

## Discussions

SONFH is a common clinical condition that often leads to collapse of the femoral head and impairment of hip joint function, resulting in high disability rates. The biological basis of SONFH is the apoptosis of osteoblasts caused by high-dose short-term or low-dose long-term glucocorticoid (GC) administration (15, 16). Additionally, abnormal osteoclast activity can lead to the loss of bone structural integrity and subchondral fractures in SONFH, but the pathological and physiological mechanisms underlying osteoclast recruitment and excessive activation in SONFH are still not fully understood. Chen et al.’s study (17) evaluated the changes in reactive oxygen species (ROS) levels and subsequent osteoclast alterations in a rat model of SONFH induced by steroids. The results indicated that the increase in ROS was proportional to the number of osteoclasts. Sun et al.’s study (18) also supported this finding. Moreover, the excessive production of ROS reduces the synthesis of vasodilators, leading to the formation of microthrombi in endothelial cells, which block local blood vessels and cause ischemic necrosis in the femoral head’s neurovascular supply area, further exacerbating bone cell damage. This study suggests that antioxidants may be a promising therapeutic approach to prevent or mitigate the progression of steroid-induced SONFH by inhibiting ROS levels and excessive osteoclast activity.

Mitochondria are highly evolved systems that govern energy production and distribution in eukaryotic cells based on specific requirements for calorie and oxygen maintenance or proliferation. In addition to regulating energy production and balancing biosynthetic precursors, mitochondria also control many other cellular parameters, including cytosolic calcium (Ca2+) levels, redox state regulation, reactive oxygen species (ROS) generation, and initiation of cell apoptosis through the activation of the mitochondrial permeability transition pore (mtPTP) (19, 20). Mitochondria are the primary source of ROS (i.e., superoxide anion, hydrogen peroxide, and hydroxyl radicals), which are generated as a result of aerobic respiration and oxidative phosphorylation (OXPHOS), reflecting an imbalance between reactive species production and antioxidant defense. When ROS species overwhelm intrinsic antioxidant capacity, oxidative stress occurs, causing damage to biomolecules in normal cells and tissues. The most common ROS-dependent injuries include impaired vascular tone and platelet adhesion, proliferation changes, gene transcription, and metabolism. Hydrogen peroxide, for instance, acts as an intracellular messenger capable of controlling cell apoptosis, senescence, and autophagy (21). Additionally, the Bax/Bak proteins in mitochondria (members of the Bcl-2 family, involved in mitochondrial outer membrane permeabilization-MOMP) or caspase activation also participate in the processes of cell apoptosis and autophagy (22). Although the cross-regulation between their involvement in cell apoptosis, senescence, and autophagy is still not fully understood and requires further research to elucidate their relationships, it is undeniable that mitochondria play a crucial role in these processes.

In our study, we selected the hormone-related gene datasets GSE123568 and GSE74089, which are associated with SONFH, from the GEO database. Additionally, we utilized the mitochondrial-related gene set from MitoCarta3.0 as the foundation for our research. The aim was to explore the relationship between SONFH and mitochondria in the context of hormone regulation. As a result, we identified seven key mitochondrial hub genes, namely BID, FTH1, LACTB, PDK3, RAB5IF, SOD2, and SQOR, which showed a high correlation with SONFH. Previously, relevant literature has demonstrated that protecting mitochondria can prevent osteonecrosis of bone cells (23). Therefore, based on our review of the relevant literature and the results of this study, we have divided the role of mitochondria in the occurrence of SONFH into two aspects. Firstly, hormone-induced activation of the mitochondrial apoptotic pathway can lead to osteocyte death and subsequently result in SONFH. The findings of Sun et al. (14) suggest that dexamethasone can induce the P53/SLC7A11/GPX4 pathway, leading to ferroptosis and consequently causing femoral head necrosis. Additionally, the studies by Zhang et al. (24) and Yao et al. (25) have demonstrated that mitigating mitochondrial apoptosis pathways by activating the Nrf2 signaling cascade can alleviate GC-induced femoral head necrosis. The above findings underscore the significant role of the mitochondrial apoptotic pathway in the occurrence of SONFH. In our study, we observed elevated expression of two genes, Bid and FTH1, in the SONFH group. Both BID and FTH1 play crucial roles in the mitochondrial apoptotic pathway. BID is involved in the Bax/Bcl2 signaling pathway, primarily mediating cell apoptosis, while FTH1 participates in the iron death pathway, inducing ferroptosis, ultimately leading to cell apoptosis. Our results once again confirm the pivotal role of the mitochondrial apoptotic pathway in the development of SONFH. Furthermore, abnormalities in mitochondrial metabolism related to reactive oxygen species (ROS) clearance and lipid metabolism may also play a crucial role in SONFH. Current literature partially supports the notion that increased ROS and oxidative stress reactions can lead to bone necrosis, with glucocorticoids (GC) contributing to mitochondrial oxidative stress reactions and metabolic dysfunction (26, 27). In our study results, we observed upregulation of genes such as SOD2, SQOR, and LACTB, which primarily regulate mitochondrial functions related to ROS and lipid metabolism. Considering these two mechanistic pathways, we hypothesize that by providing additional protection to mitochondria, it may be possible to prevent and alleviate femoral head necrosis and associated symptoms caused by GC. In the following sections, we will discuss and summarize the specific findings of this study.

Bid is a pro-apoptotic member of the Bcl-2 family, initially discovered through its interaction with pro-apoptotic Bax and anti-apoptotic Bcl-2. Bid is a 22-kDa α-helical protein that adopts the characteristic α-helical fold shared by other members of the Bcl-2 protein family. During the process of cellular apoptosis, Bid can be cleaved by caspase-8 not only during death receptor apoptotic signaling but also by other cysteine proteases, granule protease B, calpain, and tissue protease (28). Cleaved Bid translocates to the mitochondria, where it induces permeabilization of the mitochondrial outer membrane (MOMP) dependent on the pro-apoptotic proteins Bax and/or Bak, thus acting as a sentinel for protease-mediated death signaling. The cellular and molecular timeline of Bid cleavage into p7 and tBid, as well as its translocation to the outer mitochondrial membrane after apoptosis induction, has been studied in the past, and a more precise subdivision of this timeline would be highly valuable. Aberrations in BID expression or function have been implicated in various diseases, and BID’s clinical relevance extends to conditions such as cancer, neurodegenerative disorders, and autoimmune diseases. Investigating BID as a potential diagnostic or prognostic marker, as well as exploring its role in targeted therapies, holds promise for advancing personalized medicine approaches (29). Currently, research by Zaman et al. suggests that treatment with glucocorticoids (GCs) leads to an imbalance between pro-apoptotic and anti-apoptotic Bcl-2 family proteins. Specific targeting and silencing of Bid can prevent GC-induced apoptosis in human chondrocytes. Additionally, it has been found that high doses of GCs can cause oxidative stress in osteoblasts, leading to the accumulation of excessive reactive oxygen species (ROS) and increased endoplasmic reticulum stress, mitochondrial DNA (mtDNA) damage, mitochondrial dysfunction, and even significant activation of the BCL-2/BAX apoptotic pathway (30, 31). These findings pave the way for potential therapeutic strategies to rescue GC-induced avascular necrosis of the femoral head by specifically targeting the mitochondria. In our study, we found that Bid is upregulated in SONFH patients compared to healthy individuals, which may be associated with the process of osteoblast apoptosis in GC-induced femoral head osteonecrosis. Our analysis suggests that further investigation of Bid or the Bcl-2 family proteins in SONFH could provide new insights into the pathogenesis, early diagnosis, and even specific treatment of SONFH.

Mitochondrial ROS, generated as superoxide anions (O2−) during mitochondrial respiration, are eliminated by antioxidant defense mechanisms in young organisms, including superoxide dismutase 2 (SOD2), whose expression and activity decrease in aging mesenchymal stem cells along with an increase in mtROS production (32). SODs are the first line of defense against ROS. They catalyze the conversion of superoxide anions (O2−) into molecular oxygen and hydrogen peroxide; the latter is then converted to water by catalase. SODs can be used as anti-inflammatory agents, and SOD mimetics have shown potential therapeutic effects in age-related and inflammatory diseases, including neutrophil-mediated inflammation (33, 34). In humans, three forms of SOD exist: copper-zinc SOD1 and extracellular SOD3, located in the cytoplasm and extracellular compartment, respectively, and manganese SOD2, located in the mitochondria, which is essential for the elimination of superoxide radicals released primarily from mitochondrial complex III during cellular respiration, the main source of intracellular ROS (35). During osteoblast differentiation, upregulation of SOD2 is necessary to maintain mitochondrial function and osteoblast differentiation (36). It has been demonstrated that the aging of rat and human mesenchymal stem cells is associated with decreased SOD2 expression and activity and increased production of mitochondrial ROS (mtROS) (37–40). Reduced SOD2 activity and mitochondrial oxidative stress have been shown to be associated with skin and brain aging (41–43). Cellular senescence is considered a stress response typically caused by various external and internal insults, including radiation, oxidative stress, and mitochondrial dysfunction, initially identified as a highly stable cell cycle arrest (44). Recent studies have indicated the critical role of mitochondrial oxidative stress and functional SOD2 in osseointegration of implants (45, 46). Additionally, Peng et al. have shown that GC can induce osteoblast apoptosis and autophagy through the ROS/JNK/c-Jun signaling pathway, contributing to the occurrence and progression of SONFH (15), while Chen et al. (17) and Sun et al. (18) found that high levels of ROS lead to increased activity and numbers of osteoclasts, and inhibiting ROS levels and excessive osteoclast activity can prevent or alleviate the progression of steroid-induced SONFH. Therefore, further investigation of SOD2 can elucidate its role in the mechanism of femoral head ischemic necrosis, and antioxidant therapy may help prevent and control the progression of GC-induced femoral head ischemic necrosis.

LACTB is a gene that encodes a specific protein, playing a pivotal role in cellular biology. The protein encoded by LACTB is also referred to as LACTB protein and belongs to the β-lactamase superfamily (47). One of the key functions of the LACTB protein is its involvement in mitochondrial fatty acid oxidation metabolism. It possesses β-lactamase activity, enabling it to hydrolyze fatty acyl-CoA substrates within the mitochondrial membrane, thereby facilitating the generation of energy through the β-oxidation pathway. This process is crucial for maintaining cellular energy balance and survival. Furthermore, LACTB is believed to have antioxidant stress and anti-apoptotic roles. It may contribute to preserving mitochondrial function and reducing cell damage caused by oxidative stress (48). LACTB exerts its tumor suppressor function by inhibiting mitochondrial phospholipid synthesis. Downregulation of LACTB expression is usually associated with poor prognosis in these malignancies (49). LACTB has gained significant attention and research as a novel tumor suppressor. Studies have shown that LACTB plays an important role in inhibiting liver cancer (50), breast cancer (51), bladder cancer (48), and colorectal cancer (52), and downregulation of LACTB expression often indicates poor prognosis. The specific mechanisms of LACTB’s action are still under investigation, but literature suggests that LACTB exerts its anticancer effects by regulating mitochondrial lipid metabolism (53, 54). Accumulation of lipids in rabbit femoral head bone cells was studied after high-dose corticosteroid administration. Electron microscopy revealed an increase in lipid droplets within the bone cells, compressing the nucleus and leading to loss of cell membrane integrity and cell death (2). Wang et al. (55) demonstrated that corticosteroids may direct mesenchymal cells towards the adipocyte pathway rather than the osteoblast pathway. Therefore, we speculate that the dysregulation of lipid metabolism induced by corticosteroids in glucocorticoid-induced osteonecrosis of the femoral head leads to upregulation of LACTB expression.

LncRNA RAB5IF has NR_026562 (Accession number), C20orf24 (Gene symbol), Exon sense-overlapping (Classification of LncRNAs) and human chromosome 20 location. The biological functions of RAB5IF remain nebulous (56, 57). Research has shown that RAB5IF is associated with liver cancer and skeletal abnormalities, but the specific mechanisms still require further exploration. Therefore, there may be a certain correlation between the RAB5IF gene and femoral head ischemic necrosis. Future research can further explore this relationship to enhance our understanding and treatment of femoral head ischemic necrosis.

PDK3, short for Pyruvate Dehydrogenase Kinase 3, is a gene that encodes a specific enzyme. It belongs to the dehydrogenase kinase family and plays a key role in regulating the glycolytic process within the body, particularly concerning the enzyme pyruvate dehydrogenase (PDH). PDH is a crucial enzyme responsible for converting pyruvate generated from glucose breakdown into acetyl-CoA, which then enters the mitochondrial tricarboxylic acid cycle (TCA cycle) for energy production. The protein encoded by the PDK3 gene, also known as PDK3, possesses the function of inhibiting PDH activity. Specifically, the PDK3 protein reduces PDH enzyme activity by phosphorylation, thus limiting the conversion of pyruvate to acetyl-CoA. This process results in the retention of more pyruvate, preventing its conversion into energy, and thereby impacting cellular energy metabolism. The expression levels and activity of PDK3 are regulated by various factors, including physiological and pathological conditions. It plays a significant role in maintaining cellular energy balance and metabolic regulation, especially under conditions such as high-fat diets, diabetes, and certain tumors. Therefore, studying the function of the PDK3 gene and protein, as well as its expression in different physiological and disease states, is of paramount importance in understanding the regulation of energy metabolism and identifying potential therapeutic targets (58–60). In our study on SONFH, we found that the expression level of PDK3 is significantly elevated in SONFH patients. Experimental results from Damerau et al. (61) suggest that PDK3 is highly expressed in osteoarthritis, and slowing down the development of osteoarthritis can be achieved by inhibiting the expression of PDK3. Similar to osteoarthritis, SONFH also involves damage to articular cartilage and bone tissue, so this finding may provide new insights and approaches for the treatment of SONFH. In conclusion, as an important regulatory gene, PDK3 may play a significant role in the occurrence and development of femoral head ischemic necrosis. Future research will further explore the regulatory mechanisms of PDK3 and its potential applications in the treatment of SONFH.

SQOR (Sulfide Quinone Oxidoreductase) is a gene that encodes a specific enzyme, playing a pivotal role in cellular biochemistry. The enzyme SQOR, encoded by the SQOR gene, is a critical component in the sulfide metabolism pathway. Its primary function involves catalyzing the electron transfer reaction between sulfides (such as hydrogen sulfide and thiol compounds) and coenzyme Q (Coenzyme Q, also known as Ubiquinone).

The main functions of SQOR include the oxidation of sulfides to sulfite or thiol compounds, concurrently reducing coenzyme Q to its reduced form (62, 63). This reaction is of paramount importance in cellular sulfide metabolism as sulfides are toxic gases that can have detrimental effects on cells when accumulated in excess. By converting sulfides into more stable and water-soluble compounds, SQOR contributes to maintaining sulfide balance and protecting cells from the harmful effects of sulfides. Additionally, SQOR is associated with energy metabolism as coenzyme Q is a critical molecule in the mitochondrial electron transport chain, participating in ATP (Adenosine Triphosphate) energy production. Therefore, the activity of SQOR may also influence cellular energy production. In conclusion, the enzyme SQOR, encoded by the SQOR gene, plays a crucial role in cellular sulfide metabolism and energy metabolism (64). Glucocorticoids may influence sulfide metabolism and mitochondrial function by regulating the expression of the SQOR gene. Studies have found that glucocorticoids can regulate the expression of various genes, including those involved in mitochondrial biology. Hence, glucocorticoids may indirectly affect mitochondrial function and oxidative stress, leading to the occurrence of SONFH, by influencing the expression of the SQOR gene.

FTH1 is a gene that encodes a specific protein, playing a critical role in cellular biology. The protein, encoded by the FTH1 gene, is referred to as Ferritin Heavy Chain 1 and belongs to the ferritin protein family. Ferritins have a pivotal function in cellular iron storage and regulation, ensuring the efficient utilization and distribution of iron. One of the primary functions of the FTH1 protein is iron storage. Iron is an essential element required for normal cellular functioning, but excessive free iron can have detrimental effects on cells, triggering oxidative stress reactions and damaging cell structures and functions (65, 66). FTH1 accomplishes this by sequestering surplus iron ions within the core region of the protein, forming a controllable iron reservoir. This aids in maintaining stable levels of iron ions within cells, thereby preventing the accumulation of harmful free iron ions. Furthermore, FTH1 is involved in regulating cellular iron metabolism. It can release stored iron based on the cell’s demands, supplying iron for iron-dependent biochemical reactions and enzyme activities. This regulation is crucial for maintaining normal cell functions, especially in terms of systemic iron balance and red blood cell production (67). The expression of the FTH1 gene has been associated with various diseases, including tumors, cardiovascular diseases, and neurodegenerative disorders (68). Under certain circumstances, abnormal expression of the FTH1 gene may disrupt intracellular iron ion metabolism, leading to either an accumulation or deficiency of iron ions within cells. Consequently, this can impact biological processes such as cell growth, differentiation, and apoptosis. Additionally, the FTH1 gene is involved in cellular immunity, oxidative stress, and other biological processes. Research has demonstrated the significant protective role of the FTH1 gene in oxidative stress responses, as it can inhibit the generation and eliminate free radicals, safeguarding cells against oxidative damage (69). Studies have also indicated a close relationship between the Nrf2 pathway and the FTH1 gene, as the Nrf2 pathway can protect cells from oxidative stress and toxic substances by regulating the expression of the FTH1 gene (70). During hormone usage, the metabolism of intracellular iron ions may be affected, leading to either an accumulation or deficiency of iron ions. In such cases, abnormal expression of the FTH1 gene can impact the metabolism of intracellular iron ions, thereby influencing the growth and development of the femoral head and increasing the risk of SONFH (14, 71).

In our analysis of GO and KEGG, we found a close association between differentially expressed genes (DEGs) and the mitochondrial apoptosis signaling pathway. Among these DEGs, bid is a member of the Bcl-2 pro-apoptotic protein family. Several studies have shown that regulating the expression of Bcl-2 and inhibiting the expression of Bax through *in vitro* and *in vivo* experiments can prevent the death of bone cells and thus prevent osteonecrosis of the femoral head (72–74). Our research further confirms this fact, suggesting that targeted drug regulation of the Bcl-2 protein family may be an important approach for treating ischemic necrosis of the femoral head. SOD2 and SQOR are primarily involved in the clearance of superoxide radicals and sulfides during the mitochondrial redox process. If this process is impaired, excessive superoxide and sulfide can be toxic to cells, leading to mitochondrial dysfunction and ultimately inducing mitochondria-mediated cell apoptosis. Some studies have indicated that increasing the activity of SOD can help counteract the progression of femoral head necrosis in the context of apoptosis mediated by the Bcl-2 protein family. Therefore, SOD and SQOR may hold significant value in inhibiting the progression of femoral head necrosis (75), requiring further exploration. Research has shown a close relationship between the Nrf2 pathway and the FTH1 gene. The Nrf2 pathway can protect cells from oxidative stress and toxic substances by regulating the expression of the FTH1 gene. Activation of the Nrf2 pathway promotes the expression of the FTH1 gene, increasing the storage and transportation capacity of intracellular iron ions, reducing their accumulation, and protecting cells from oxidative stress and toxic substances. Additionally, the expression of the FTH1 gene can also influence the activation of the Nrf2 pathway. Studies have indicated that the expression level of the FTH1 gene can impact intracellular oxidative stress, thereby affecting the activation of the Nrf2 pathway. Abnormal expression of the FTH1 gene may increase intracellular oxidative stress, inhibiting the activation of the Nrf2 pathway and increasing the risk of cellular damage from oxidative stress and toxic substances. Yao et al. (25) demonstrated the potential of FTH1 in treating femoral head necrosis by using the PTEN inhibitor VO-OHpic to activate Nrf2 signaling and inhibit the mitochondrial apoptosis pathway, thereby alleviating GC-related dysfunction of endothelial progenitor cells and femoral head necrosis. As for the relationship between PDK3, RAB5IF, LACTB, and ischemic necrosis of the femoral head, there is currently no relevant literature reporting on it. However, these three genes are closely associated with metabolism, cell membrane stability, and cell apoptosis. Further research on them may help reveal the underlying mechanisms of femoral head necrosis.

The PPI network demonstrated the presence of relevant genes in the BCL-2/BAX apoptotic pathway and the ROS/JNK/c-Jun signaling pathway, indicating the significant role of cell apoptosis in SONFH. In terms of immune cell infiltration, we found a significant increase in the infiltration of T cells, CD8 T cells, cytotoxic lymphocytes, monocytes, neutrophils, endothelial cells, and fibroblasts in the SONFH group compared to the non-SONFH group. This finding is consistent with the study by Jiang et al. (76), who also identified neutrophil percentage as an independent protective factor for SONFH. Although the specific underlying reasons still require further investigation, the results of immune cell infiltration once again validate the value of our study. Finally, the predictive model constructed using these seven genes exhibited good diagnostic performance with an AUC of 0.93. This indicates that these seven genes may have good diagnostic value and provides a solid foundation and confidence for our future research endeavors.

## Conclusions

There is a close relationship between SONFH and mitochondria. SONFH is a skeletal disorder that is influenced by multiple factors, with hormones being one of the important contributors. Hormones participate in the occurrence and development of SONFH through various pathways, including the impact on mitochondrial function.

Mitochondria are vital organelles within cells, serving as the primary site for cellular energy metabolism and participating in various biological processes such as cell apoptosis and calcium ion regulation. Research suggests that abnormal mitochondrial function may be associated with the occurrence and development of SONFH. Hormones can be involved in SONFH by influencing mitochondrial function. For instance, glucocorticoids can affect the stability and permeability of the mitochondrial membrane, leading to mitochondrial dysfunction and cell apoptosis, thereby promoting the development of SONFH. Moreover, some studies indicate that abnormal mitochondrial function may also be related to the treatment outcomes of SONFH. For example, certain drugs can improve the symptoms and prognosis of SONFH by regulating mitochondrial function.

In our study, we further confirmed the overexpression of BID (Bcl-2 protein family) in SONFH and its close association with mitochondria-mediated cell apoptosis. We also discovered the upregulation of SOD and SQOR in SONFH and explored the relationship between genes such as PDK3, RAB5IF, and LACTB and SONFH, providing directions for our future research.

In the future, we can: 1.Further investigate how glucocorticoids specifically affect mitochondrial function, oxidative stress, cell apoptosis, and energy metabolism to uncover the pathophysiological processes of SONFH. 2.Explore mitochondrial-targeted treatment strategies, such as antioxidants, mitochondrial protectants, and energy metabolism regulators, to improve patient prognosis and reduce the incidence of SONFH. 3.Investigate whether other hormones, such as sex hormones and growth hormones, are also involved in mitochondrial dysfunction and the pathogenesis of SONFH, to provide a more comprehensive understanding and treatment strategies.

In conclusion, there is a close relationship between SONFH and mitochondria. Through in-depth research on the relationship between SONFH and mitochondria, we can gain a better understanding of the pathogenesis of this disease and lay the foundation for the development of new treatment strategies.

## Data availability statement

The original contributions presented in the study are included in the article/**Supplementary Material**. Further inquiries can be directed to the corresponding author.

## Author contributions

ZM: Writing – original draft. JS: Writing – original draft. QJ: Conceptualization, Writing – original draft. YZ: Writing – review & editing. HJ: Writing – original draft. PS: Writing – original draft. WF: Writing – review & editing.
